# Effective Management of a Tooth Eruption Bulge: A Comprehensive Case Report

**DOI:** 10.7759/cureus.65271

**Published:** 2024-07-24

**Authors:** Kashish C Kalose, Aakriti Chandra, Nilima R Thosar, Meenal S Pande, Himani Parakh, Neha Pankey

**Affiliations:** 1 Pediatric and Preventive Dentistry, Sharad Pawar Dental College and Hospital, Datta Meghe Institute of Higher Education and Research, Wardha, IND; 2 Pediatric and Preventive Dentistry, Sharad Pawar Dental College And Hospital, Datta Meghe Institute of Higher Education and Research, Wardha, IND

**Keywords:** successor teeth, eruption bulge, primary teeth, traumatic injuries, pediatric dentistry

## Abstract

An eruption bulge is a swelling or an enlargement below the gumline due to the eruption of a tooth. Radiographic images would also illustrate the fact that there may be the presence of a successor tooth (unerupted or unimpacted) under the bulge of the gingiva. Teething refers to the sequence of growth and appearance of the primary as well as permanent teeth within the oral region that varies according to age. A tooth eruption bulge is a lump that occurs due to the eruption of the teeth in the gum tissue. The appearance of the bulge varies according to the amount of fluid or blood present in this engorged organ. It is important to focus on the fact that the dentist will always be able to notice any changes and solve the issue when it comes to the check-ups, which should take place on a regular basis. Parents need to be aware of the possible adverse effects of trauma on primary incisors on the eruption as well as the health of its permanent successors. They need to watch and tell about any oral changes or conditions their child may have, like pain, color change, and swelling as this may tell of something negative to their dentition. Such a condition can only be managed using a multi-disciplinary team that includes a pedodontist, an orthodontist, and in severe cases an oral surgeon who will have to deal with the long-term sequelae of dental trauma. This is why it is necessary to educate them on these matters because then they can be proactive.

## Introduction

A dental eruption bulge occurs due to an erupting tooth causing its localized swelling or protrusion on the gums. Except for the area's increased size, the surrounding gingiva has a typical appearance in terms of color and texture. Beyond body weight and height, the eruption date of the first tooth is an obvious indicator of overall physical development. Insights regarding the child's dietary status, social background, and possible underlying medical issues are also provided [1].

Primary and permanent teeth erupt in the oral cavity over an extensive age group. Standards for appropriate eruption usually take into consideration a variety of factors, including individual features, sexual orientation, race, and ethnicity, all of which might influence tooth eruption [2-4]. In children between the ages of two to four years and nine to 10 years, one of the main causes of delayed tooth eruption is traumatic dental injury [5-7]. Damage to the primary teeth is associated with several issues with the dental pulp, including root resorption, cysts, ankylosis, pulp canal closure, and pulp death. Pulp necrosis of the primary tooth also causes delayed eruption and the development of eruption bulges [8,9]. Although eruption bulges are usually thought to be a natural aspect of the eruption process, their appearance might occasionally worry medical professionals and parents due to the discomfort they may cause and the possibility of a false diagnosis. Knowledge of the etiology, clinical features, and management of eruption bulges enables dental practitioners to appropriately manage their patients’ concerns.

The following case describes a case of traumatic dental injury (TDI) to a deciduous tooth of a nine-year-old, leading to a tooth eruption bulge of a permanent tooth, and emphasizes the management of the condition according to current literature available. The goal is to draw attention to the fact that diagnostic work is necessary and relevant and also define how to cope with this frequent dental occurrence with the process of eruption of new teeth. This will highlight the potential benefits and effectiveness of less invasive approaches in treating tooth eruption bulges.

## Case presentation

**Figure 1:**
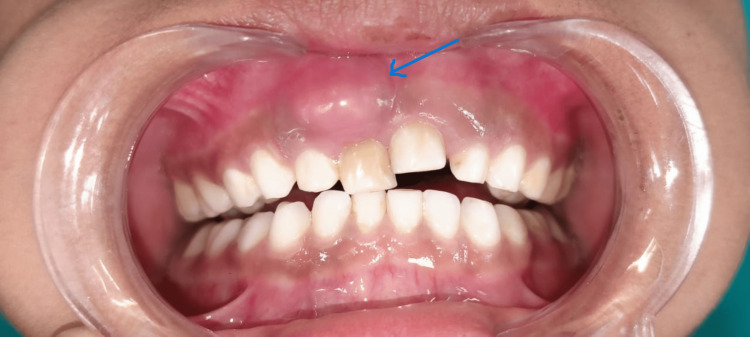
Swelling seen with right maxillary central incisor with discolored crown.

A nine-year-old male patient reported to the outpatient department in Sharad Pawar Dental College and Hospital, Sawangi (Meghe), Wardha, India, with the chief complaint of swelling of the upper front region of the jaw (Figure 1). 

**Figure 2:**
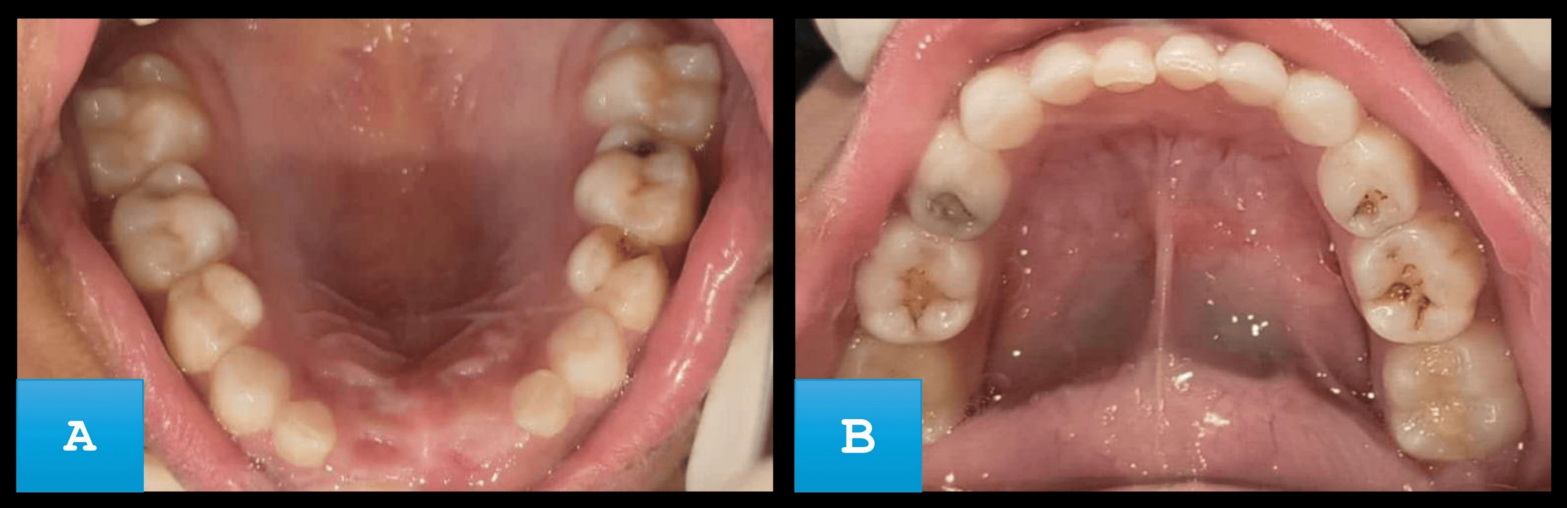
(A) Intraoral clinical photograph of maxillary arch showing pit and fissure caries with left maxillary molar. (B) Mandibular arch showing multiple carious teeth.

The swelling was not associated with any kind of pain; however, it was associated with occasional discomfort during mastication. The child has a prior traumatic dental injury in the same area that occurred due to a fall while playing one year ago. On extraoral examination, it was observed that there were no signs of facial asymmetry, bruising, or visible scars on the lips. Intraoral examination revealed a circular swelling of approximately 2 × 2 cm. The swelling matches the color of the gingiva and was located on the buccal aspect of the gingiva with respect to tooth number 51. The swelling extended from the mesial side of tooth number 51 to the labio-distal aspect of tooth number 52. While examining the hard tissue the swelling appeared to be hard and non-tender with no sign of discharge or drainage. Several carious teeth were seen in the maxillary and mandibular arch (Figure 2).

**Figure 3:**
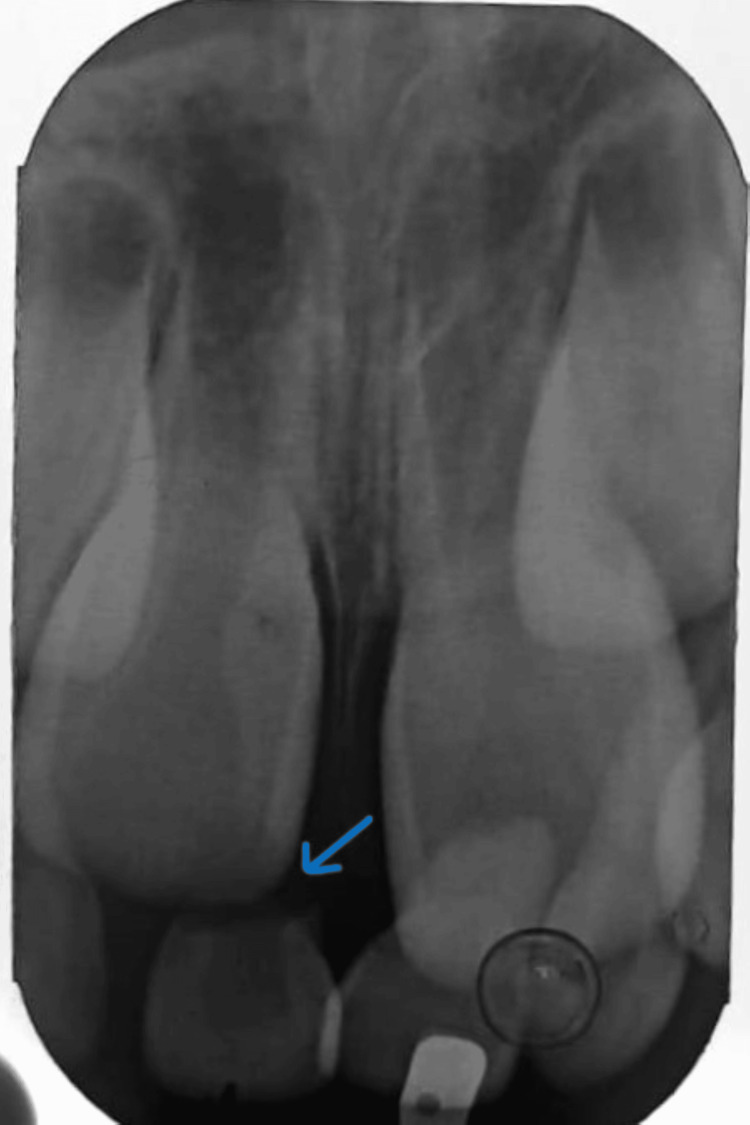
Radiograph of maxillary anterior region showing, primary maxillary right central incisor with root resorption.

There was crown discoloration with no mobility of the maxillary right primary central incisor. No increase in the dimension of the alveolar bone was seen. A radiographic examination was carried out on the patient which included an intraoral periapical radiograph (Figure 3).

An observation was made indicating the presence of a retained primary maxillary right central incisor exhibiting complete root resorption. Further observations include a mesial inclination or subluxation of the left maxillary primary central incisor. According to Nolla's stage of root development classification, the maxillary permanent teeth in the radiograph exhibited stage six [10]. The provisional diagnosis was a gingival abscess. But, upon further examination, it was determined that the swelling was due to a tooth eruption bulge, accompanied by a retained primary maxillary right central incisor that had undergone complete root resorption. Initially, the first step undertaken was to treat the affected tooth along with swelling in the gum tissue, followed by planning complete dental rehabilitation for teeth affected with dental caries. Removal of discolored deciduous central incisors was essential to create room for permanent teeth to emerge. The procedure was explained beforehand to the patient and after getting proper consent, the following treatment was carried out.

**Figure 4:**
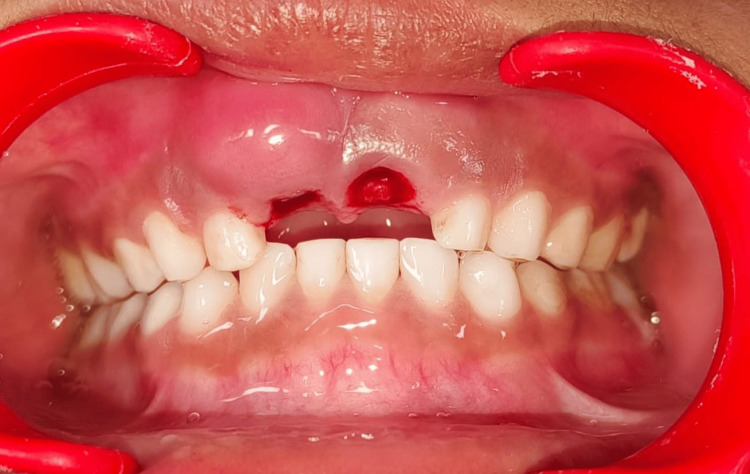
Clinical photograph showing maxillary anterior region after extraction.

Local anesthesia was administered with 2% lignocaine hydrochloride with adrenaline (at a concentration of 1:2,00,000) to anesthetize the region surrounding the tooth. Topical 5% lignocaine gel was applied prior to injection to enhance comfort. Infraorbital nerve block and nasopalatine nerve block were given. Forceps designed for the maxillary anterior region were utilized to securely hold the tooth. Efforts were made to avoid forceful and excessive movements in order to prevent any potential damage to the surrounding tissues or the development of successor teeth underneath. Following the extraction of teeth, the socket is examined for any remaining tooth or bone fragments. A sterile gauze was applied with pressure to achieve the hemostasis (Figure 4).

**Figure 5:**
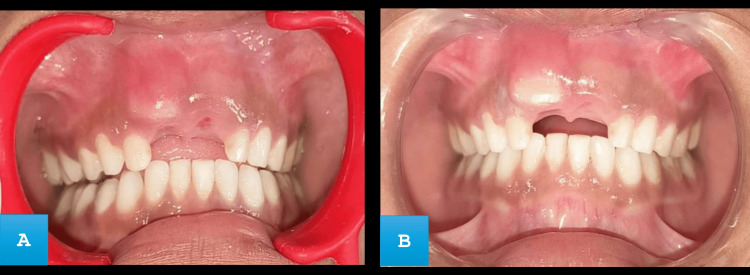
(A) Intraoral frontal view showing reduced swelling at one week. (B) Erupting tooth seen at second week.

Post-operative instructions were provided. The follow-up appointment was made after the first and second to monitor the healing progress, evaluate the growth and emergence of the permanent successor tooth, as well as discuss preventive measures for any remaining carious teeth. After one week (Figure 5A), the swelling resulting from the bulge had subsided, and by the second week (Figure 5B), a permanent maxillary central incisor had emerged without any pathological changes.

**Figure 6:**
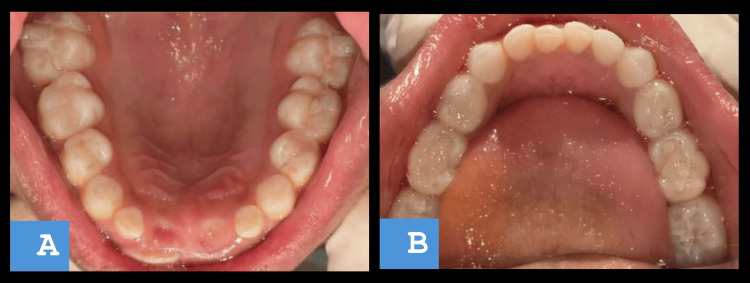
(A) Postoperative images of restoration with maxillary molar. (B) Rehabilitation done with caries removal and sealant application in the mandibular arch.

After proper healing of the affected site, the treatment for the remaining teeth was planned in the forthcoming days (Figure 6).

## Discussion

Dental trauma in deciduous teeth occurs more frequently between the ages of two to three years old. At this age, motor coordination is in the process of formation and children of both sexes begin to walk [11]. Incisors are more susceptible to fracture in TDI [12]. However, it should attract enough attention, as the absence or improper development of maxillary incisors in the form of unerupted teeth negatively affects facial aesthetics, which can lead to self-consciousness and difficulties in socializing in children [13]. Traumatic primary tooth injuries have a great probability of negatively influencing the growth of the ensuing permanent teeth. This is because follicles of the permanent successors that are still growing are anatomically close to the root apices of primary teeth [14-16]. Pulpal necrosis in certain cases can displace or restrict the eruption of a permanent tooth. Timing of intervention is important to prevent orthodontic forces to force out impacted incisors and prevent further orthodontic problems [17-19]. Delayed eruption may occur when the permanent bud has changed its position or when tissues covering the permanent tooth are abnormal if they are fibrous or thick while the tooth has been damaged in any way that results in over-growth of the connective tissues over the erupting tooth. The intraoral periapical radiograph is highly recommended to evaluate the involved tooth's morphology or surrounding bone [20]. Even though surgery may be necessary in certain instances, teeth with immature roots retain the capability to shift and eventually erupt [21].

In this particular case, a non-surgical treatment plan was adopted in which the over-retained primary incisor was extracted, the impacted permanent incisor was surgically exposed. The patient was observed in follow-up visits to assess the progress followed by complete mouth rehabilitation. The possible impacts of injury to deciduous teeth on the emergence and growth of permanent replacements were explained to parents. Symptoms such as soreness, discoloration, and other causes that warn a developing issue, signify that some harm might exist. The radiographs were used to ascertain the early developmental abnormalities and allowed the prevention or the behest minimization of things that could lead to or aggravate additional serious difficulties. Regular dental visits are also critical since they monitor the diseased development of teeth and can resolve any issue quickly. A multidisciplinary approach severely must be adopted to ensure long-term complications are managed properly. Various other conservative treatments such as simple observational techniques, and non-surgical interventions can be done by frequently applying topical anesthetics and analgesics to manage discomfort. The use of a soft tissue laser can be effective since the patient at times does not require intervention when they suffer from minor symptoms, no sign of infection, or significant discomfort.

## Conclusions

Sequential and timely eruption of teeth helps in a child’s overall development. Trauma to primary teeth can have a detrimental effect on the eruption of subsequent permanent teeth, with the central incisor roots being the most vulnerable. The natural eruption process of the permanent successor can be disrupted by the defect, and it may be delayed or entirely canceled. Providing such information to parents will alert them to the potential damaging effects on permanent teeth and reinforce the need for adequate and timely treatment and follow-up. This will allow parents to be better informed about the need for timely dental care and the need to be actively involved in the child’s dental health management.
